# Seven-year evaluation of idiopathic multiple retinal pigment epithelium detachments

**DOI:** 10.3205/oc000049

**Published:** 2016-10-05

**Authors:** Pawel Krasnicki, Diana Dmuchowska, Ewa Zukowska, Zofia Mariak

**Affiliations:** 1 Department of Ophthalmology, University Teaching Hospital of Bialystok, Poland

**Keywords:** OCT, multiple retinal pigment epithelium detachments, RPE detachments, multiple PEDs

## Abstract

**Objective:** To analyze a 7-year natural history of idiopathic multiple retinal pigment epithelium (RPE) detachment based on optical coherence tomography (OCT) images.

**Methods:** Case report. SD-OCT imaging (Topcon 3D OCT-1000).

**Results:** A 55-year-old female presented with three foci of pigment epithelium detachment (PED) in her left eye. Her past ophthalmic and medical history was uneventful. The patient’s visual acuity in both eyes was 1.0 throughout the follow-up period. Fluorescein angiography images are shown. Corresponding OCT scans illustrate natural history of the PED foci and retinal structure of the left eye. PED number, location, shape, size and morphology were analyzed. Initially, the dimensions of PEDs were stable, but then a tendency to fluctuate or flatten was observed. Eventually, the lesions have resolved completely. Apart from the detachments, no other structural abnormalities of the retina were found. No PEDs, but sub-, para- and perifoveal RPE protrusions and defects were detected in the right eye.

**Conclusions:** In the hereby presented case:

1. OCT proved to be suitable for diagnosis and monitoring of multiple PEDs.

2. Multiple idiopathic PEDs without involvement of the fovea were asymptomatic and regressed spontaneously. As such, they did not require any treatment. However, they were monitored due to potential risk for choroidal neovascularization or serous retinal detachment.

## Introduction

We present a 7-year natural history of idiopathic multiple retinal pigment epithelium (RPE) detachment assessed by optical coherence tomography (OCT). To the best of our knowledge, this is the first OCT-based study documenting the evolution of PEDs in such long perspective. Furthermore, we hypothesize on potential origin of the lesions.

## Case description

**Figure 1:**
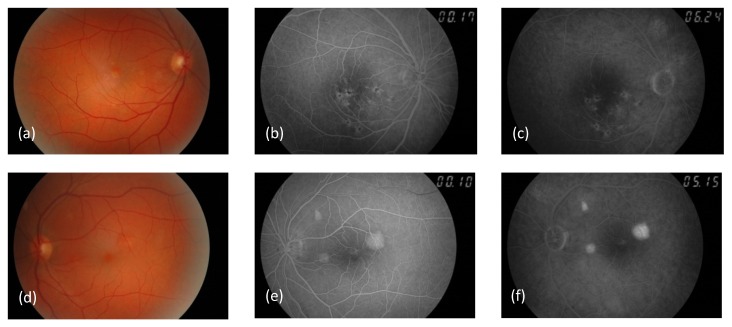
Right (a–c) and left (d–f) eye. Color photographs of the fundus (a, d) , early- (b, e) and late-phase (c, f) fluorescein angiograms

A 55-year-old female presented with three foci of pigment epithelium detachment (PED) in her left eye, found accidentally during a routine examination. Past ophthalmic and medical history of the patient was uneventful; she had no history of systemic diseases (e.g. hypertension, collagen vascular diseases), topical and oral corticosteroid administration. The patient’s visual acuity in both eyes was 1.0 throughout the follow-up period. The results of the Amsler test were positive for the right eye and negative for the left eye. Fundoscopy revealed pigment clumpings surrounded by a yellow halo in the macular region of the right eye, and slightly elevated yellowish round PEDs in the left eye (Figure 1 a, d (Fig. 1)). Otherwise, the results of the ophthalmic examination were normal.

**Figure 2:**
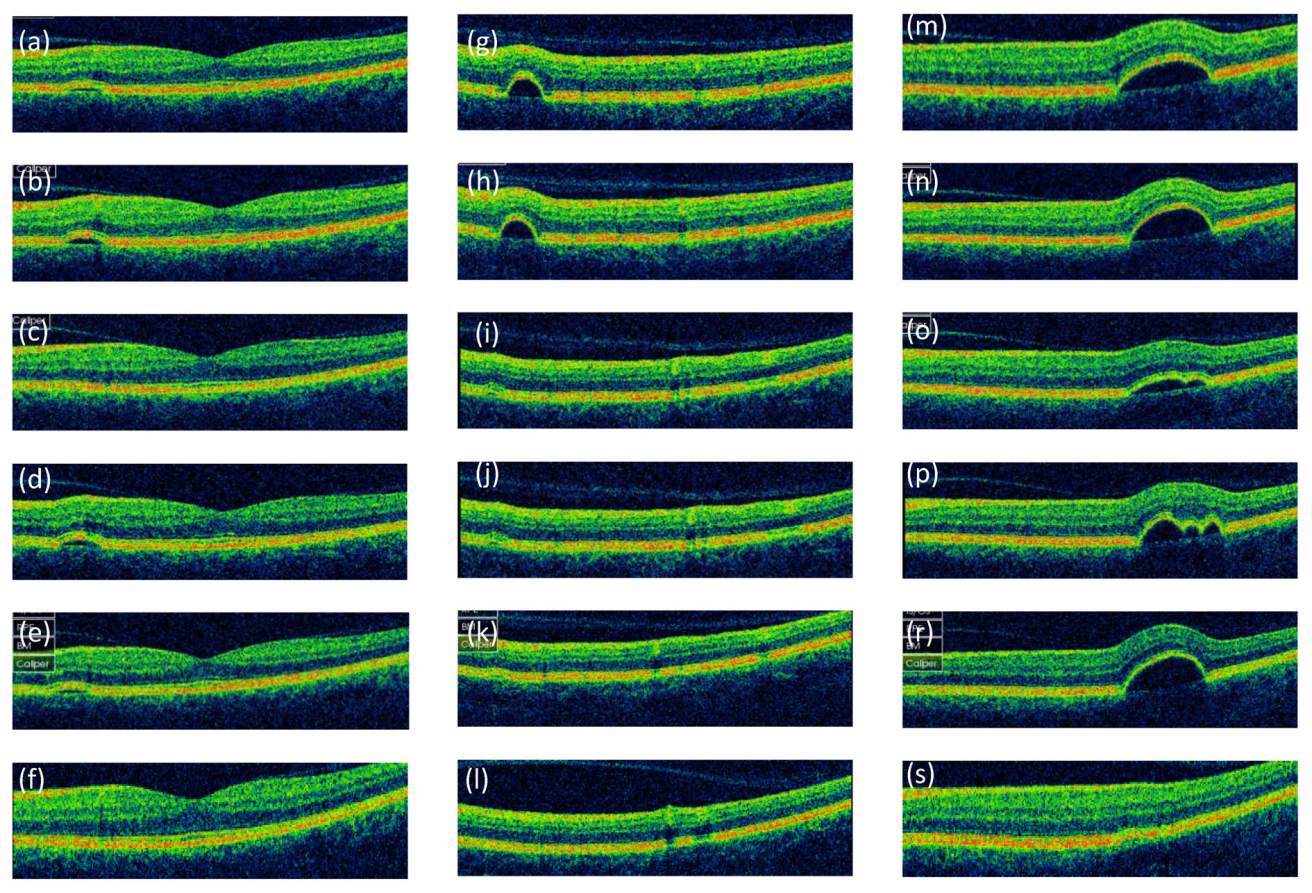
Serial horizontal OCT scans of the left eye through the RPE detachments illustrating their natural history Three dome-shaped RPE detachments were found, 1,670 µm from the fovea at 9 o’clock (left panels), 3,200 µm from the fovea at 10:30 o’clock (middle panel), and 750 µm from the fovea at 2 o’clock (right panel). The images were taken at the baseline, as well as after one, two, three, four and seven years of follow-up (from top to bottom, respectively). All the PEDs had round basis with diameters ranging from 498 to 593 µm (left panel), 423 to 486 µm (middle panel) and 1,144 to 1,225 µm (right panel). The height of the elevations varied, reflecting their flattening and complete resolution, and ranged from minimal to 63 µm (left panel), minimal to 135 µm (middle panel) and 106 to 235 µm (right panel). The presence of the detachments was not associated with fluid accumulation between RPE and neurosensory retina. The RPE layer within the detachment was continuous and showed no defects. The thickness, structure and foveal contour of the overlying retina were normal.

Fluorescein angiography performed at the baseline demonstrated multifocal, irregularly distributed fluorescence-blocking lesions surrounded by a halo of window defect in the right eye (Figure 1 b, c (Fig. 1)), as well as three foci of dye pooling without active leakage in the left eye (Figure 1 e, f (Fig. 1)). A 7-year natural history of these PED foci and the retinal structure of the left eye are illustrated on OCT raster scans (Figure 2 (Fig. 2)). OCT enabled us to detect the PEDs, conduct a differential diagnosis and monitor the PEDs. Furthermore OCT is an adequate method to analyze retinal and RPE lesions. Number, location, shape, size and morphology of the PEDs in the left eye were analyzed. Aside from the detachments, no other retinal structural abnormalities were detected. Initially, the dimensions of the PEDs were stable, but then a tendency to fluctuate or flatten was observed. All three foci resolved completely and only residual minor RPE defects were observed at the end of the 7-year follow-up. While no PEDs were found, sub-, para- and perifoveal RPE protrusions and defects were detected in the right eye. Otherwise, the retinal structure was normal.

## Discussion

PED is a nonspecific finding. Differential diagnosis should include, among others, central serous chorioretinopathy (CSR), age-related macular degeneration (AMD), polypoidal choroidal vasculopathy, hypertensive chorioidopathy, choroidal tumors [1] and Vogt-Koyanagi-Harada disease [1], [2].

Currently, CSR is believed to be initiated by RPE dysfunction resulting from primary pathology within the Bruch’s membrane, choriocapillaris, or both [3], [4]. Bilateral asymmetric involvement has been reported in up to 40% of the cases [5]. It is more common among older patients (>50 years of age) [6]. Older age is also associated with a chronic course of the disease [5], [6]. Concomitant PEDs may be found in 5% to 63% of the cases [4]. In the hereby presented case, OCT scans showed predominantly dome-shaped, smooth and transparent PEDs; such presentation is consistent with the description published by Lumbroso et al. [7]. Our findings were specific for type II CSR according to the classification presented by Vukojević et al. [8]. It is characterized by the accumulation of liquid under localized elevation of the pigment epithelium, in contrast to the more common type I CSR, where fluid accumulates under the neurosensory retina.

Multiple PEDs in the left eye of our patient might represent an active, chronic or recurrent CSR, as reported previously by Wang et al. [3], Song et al. [9], and Bandello et al. [10]. However, multiple PEDs do not necessarily develop secondarily to CRS; they may also precede bilateral CRS with serous retinal detachment, as reported by Wang et al. [3] and Bandello et al. [10]. Interestingly, both regression and progression of PEDs were observed in our patient during the 7-year follow-up period. Katsimpris et al. [11] used fluorescein angiograms to follow-up a severe active bilateral CRS for a period of 16 years, and also observed a chronic course of the disease.

Resolution of CSR is often followed by local atrophy of RPE and pigmentary changes in the macula, which may resemble AMD [12]. Gupta et al. [13] studied three dimensional single-layer RPE map on SD-OCT and documented presence of RPE bumps in 94% of asymptomatic contralateral eyes of patients with idiopathic CSR. We assumed that pigment mottling of the right eye represented residual abnormalities resulting from previously resolved asymptomatic episodes of CSR. These lesions did not progress in size and severity during the follow-up period which excluded AMD as the diagnosis. Furthermore, no concomitant symptoms specific for AMD, such as drusen, geographic atrophy, subretinal neovascularization, intraretinal and subretinal fluid accumulation were noted. On fluorescein angiography, the lesions present in the left eye our patient showed uniform hyperfluorescence at early phases, and well-demarcated pooling of the dye within constant borders at late phases, which is specific for typical serous PEDs. In contrast, AMD is often associated with presence of fibrovascular or hemorrhagic PEDs.

Based on the patient’s medical history, a detailed examination of the fundus and the analysis of OCT and FA images, we excluded with high probability less frequent potential causes of PEDs, such as hypertensive chorioidopathy, choroidal tumors [1] and Vogt-Koyanagi-Harada disease [2].

Further long-term monitoring of the patient will determine if the resolution observed during the course of idiopathic recurrent CSR was permanent or only temporal.

## Conclusions

We present a case of multiple RPE detachments in the course of idiopathic CSR without involvement of the fovea. These lesions, not connected with systemic diseases or drugs administration, were asymptomatic and regressed spontaneously. As such, they do not require any treatment. However, they should be monitored due to potential risk for choroidal neovascularization [3] or serous retinal detachment [10].

## Notes

### Competing interests

The authors declare that they have no competing interests.
